# Chicago Medical Society

**Published:** 1879-07

**Authors:** Roswell Park

**Affiliations:** President, in the chair.


### ARTICLE XI.

CHICAGO MEDICAL SOCIETY. Regular meeting May 5. Reported by ROSWELL PARK, M D. DR. ANDREWS, President, in the chair.

DR. F. C. HOTZ reported the following case of Traumatic Aneurism of the Eyelid : The doctor had performed an operation for trichiasis upon the upper lid of a young lady. For the purpose of transplanting the false eyelashes, an incision was made along the free edge of the lid. On the third day the sutures were removed, and the wounds were found to be healed by first union, except a small gap in the center of the free edge. Ten days after the operation, a profuse hæmorrhage occurred, the source of which proved to be a small aneurism protruding from that gap at the center of the edge of the lid. The aneurismal sac was excised, and pressure bandage applied. The wound then closed up by firm cicatrization.

DR. F. C. HOTZ also reported the case of chloroform asphyxia described in an article in the June number of the JOURNAL AND EXAMINER. In the discussion which followed this report, the President said he recollected an analogous case of an intemperate man with delirium tremens, who refused all food and medicine, and desperately resisted all attempts to administer either. He directed the administration of sufficient chloroform to at least render possible the exhibition of morphia. (This before the days of hypodermic medication.) The plan succeeded admirably, till suddenly respiration ceased, the usual symptoms supervened, and the patient died in spite of all efforts to resuscitate.

DR. HOLMES said that personally he had discontinued the use of chloroform some nine years ago, except in the case of young children, although his assistants often administered it. He had been unfortunate enough to have seven or eight deaths, from its use, on the table. It was now a number of years since he adopted the method of raising the feet as an aid to restoration of vitality. Alluding to the fact that sometimes alarming symptoms came like a shot, while at other times anæsthesia was almost completely recovered from before they were noticed, he thought it essential to safety to carefully watch these cases until complete consciousness had returned.

DR. BOGUE, during fourteen years' practice, had seen four cases die from the effects of chloroform; and each of them while it was being administered. He considered that in two cases of the four, death was simply inevitable from idiosyncrasy on the part of the patient; in the other two this was not so certainly the case. He was positive that in one case he observed cardiac pulsations for fully twenty minutes after cessation of respiration; artificial respiration was kept up as long as the pulse beat.

DR. F. H. DAVIS deemed it likely that in these cases of chloroform asphyxia it was as often a poisoning from carbonic dioxide on account of deficient æration of the blood, as from the pure paralysis of respiration caused simply by the chloroform.

DR. R. PARK alluded to the contrary statements laid before the students in the text books; one author stating that chloroform kills by paralyzing the cardiac centers, another that it paralyzes the respiratory centers; the former being the most common statement, but made with many modifications. He had learned of cases which justified each assertion, and wished that such contradictory statements might be either reconciled or modified. He referred to an admirable paper by Dr. Chisholm, of Baltimore, giving a concise account of the dangers from the use of chloroform, in which he enumerated them as follows. First, pure carelessness in administration, giving the vapor too strong, or not watching the patient attentively. Second, reflex spasm of the laryngeal apparatus causing strangulation; the remedy for which is simple. Third, inattention on the part of the one who administers the anæsthetic, and inadequate or improper means of administration. To use a thick towel saturated and folded around the face should be regarded as a criminal procedure. Fourth, the emesis caused frequently by anæsthetics has been more than once the real cause of death which was attributed to the latter; inasmuch as some of the contents of the stomach may be drawn from the pharynx into the trachea by violent inspiratory effort, if the patient be allowed to remain on his back. Hence the caution to turn the patient on the side as soon as there is the slightest sign of nausea. Fifth, failure to produce perfect and profound anæsthesia; since any condition short of this leaves the cardiac and respiratory centers exposed to inroads from peripheral irritation by which their function may be suddenly arrested. In this way may be accounted for those deaths that have occurred under anæsthesia for trivial operations, as well as those during more serious operations, where the timidity or anxiety of the surgeon causes him to stop the inhalation before the period of safety has been reached. Sixth, excessive administration; and seventh, the only legitimate and rarest cause of death from anæsthetics, that unknown condition called idiosyncrasy.

DR. PARK said he had ventured to give chloroform in cases of acute rheumatic endocarditis for the purpose of moving and cleansing the patient. He commended in the highest terms the method of administering the drug which he had learned from Dr. Bogue, of giving it drop by drop, from a dropping bottle, upon a single thickness of cloth held just in front of the patient's nose. This ensured free dilution of the vapor, and was, he thought, the nearest approach to perfect safety. In this way he had anæsthetized with two grams of the drug.

Alluding to the use of amyl nitrate with chloroform, he said he was now using it so, using about 1 part of the former to 300 parts of the latter. Judging from a few trials, he was inclined to think that more prostration and slower recovery followed the use of the mixture than the administration of the pure drug.

The discussion having terminated, DR. ANDREWS exhibited an oxalate of calcium calculus, about a line in diameter, which would have passed through the urethra without trouble, had not a congenital stricture of the meatus detained. It was readily removed by a simple incision and the obstruction to the flow of urine removed.

DR. F. H. DAVIS gave a brief account of a patient suffering from caries of the spine, who coughed up a little foreign body which was sent to him for examination. Under the microscope it exhibited the characteristics of true bone, and was doubtless discharged fram a carious bone into the lung by the destructive processes, whence it was expectorated without difficulty.

---

MEETING MAY 19, DR. ANDREWS, President, in the chair.

DR. ANDREWS read a paper entitled, "The Corn Doctor's Progress," which appears in this number. It was received with interest, but elicited no particular discussion.

---

CITRATE OF CAFFEIN AS A DIURETIC IN CARDIAC DROPSY. Shapter. (*Practitioner*, *Jan.*, '79.)

Professor Gubler first called attention to the diuretic properties of citrate or hydrobromate of caffein, in doses of 0.25 to 0.50, as inducing abundant and immediate diuresis in cases of cardiac dropsy, when given either subcutaneously or by the mouth. S. confirms this observation by the report of four cases, in each of which the flow of urine was more than doubled by its use.

The reporters experience with it leads him to assign to it a special position as a cardiac diuretic in those cases of cardiac disorder where muscular embarrassment and neurosal incöordinate cardiac action (the indications of progressive mural decay) clinically forbid the administration of "tonic" doses of digitalis which may relieve the stagnating pressure of venous blood in the right side of the heart.

According to Binz, caffein is a diuretic or excitor of venal secretion, and it increases the heart's action either by direct stimulation to the organ itself, or by the arteries which it excites to contraction.

Practically, then, where a dilated, feeble heart contracts irregularly and is undergoing progressive mural degeneration, and where dropsy as the result is the most obvious symptom, S. recommends the above salt as the very best remedy offered

