# Correction

**Published:** 1879-09

**Authors:** 


### CORRECTION.

In our report of the meeting of the Chicago Medical Society, in the last number of the JOURNAL AND EXAMINER, we inadvertently did Dr. E. L. Holmes an injustice, for which we hasten to make reparation. In the discussion on chloroform asphyxia, he is represented as stating that he had been so unfortunate as to have seen seven or eight deaths on the table from its use. It should have read *apparent* deaths — *i. e.*, most alarming symptoms: syncope, etc.; but the Doctor has never had a *fatal* case of chloroform poisoning. He mentioned also that evening that he had had *one* fatal result from the administration of ether, in 1876; which was duly reported in the files of the *Chicago Medical Journal.*

He also alluded further to the method of resuscitation by depressing the head or elevating the feet — a method which was original with him, and which he was the first in this country to apply, although the distinguished Nelaton also resorted to it, and is probably entitled to the honor of priority in its employment.

We trust that this correction may meet the eye of every one who may have received a wrong impression from the report alluded to.

